# Prenatal Isolated Ventricular Septal Defect May Not Be Associated with Trisomy 21

**DOI:** 10.3390/jcm3020432

**Published:** 2014-04-23

**Authors:** Ori Shen, Sari Lieberman, Benjamin Farber, Daniel Terner, Amnon Lahad, Ephrat Levy-Lahad

**Affiliations:** 1 Department of Obstetrics and Gynecology, Shaare Zedek Medical Center pob 3235, Jerusalem 91031, Israel; E-Mail: terner10@netvision.net.il; 2 Department of Pediatric Cardiology, Shaare Zedek Medical Center pob 3235, Jerusalem 91031, Israel; E-Mail: noralia@walla.com; 3 Genetics Unit, Shaare Zedek Medical Center, Jerusalem 91031, Israel; E-Mails: sari@szmc.org.il (S.L.); lahad@szmc.org.il (E.L.-L.); 4 Department of Family Medicine, Hebrew University, Jerusalem 91120, Israel; E-Mail: genetics@szmc.org.il

**Keywords:** congenital heart disease, prenatal diagnosis, trisomy 21, ventriculoseptal defect

## Abstract

The aim of this study was to examine if isolated fetal ventricular septal defect (VSD) is associated with trisomy 21. One hundred twenty six cases with prenatal VSD diagnosed by a pediatric cardiologist were reviewed. Cases with known risk factors for congenital heart disease, the presence of other major anomalies, soft signs for trisomy 21 or a positive screen test for trisomy 21 were excluded. Ninety two cases formed the study group. None of the cases in the study group had trisomy 21. The upper limit of prevalence for trisomy 21 in isolated VSD is 3%. When prenatal VSD is not associated with other major anomalies, soft markers for trisomy 21 or a positive nuchal translucency or biochemical screen, a decision whether to perform genetic amniocentesis should be individualized. The currently unknown association between isolated VSD and microdeletions and microduplications should be considered when discussing this option.

## 1. Introduction

Heart anomalies are the most common congenital defect (CHD) with an incidence of 2.5–9:1000 live births [1,2,3] and are associated with an increased risk for chromosomal anomalies. The degree of risk and the type of genetic abnormality are related to the nature of the cardiac anomaly. Ventricular septal defect (VSD) is the most common CHD, comprising 25%–32% of those diagnosed during the first year of life [4,5]. Detection rates in gestation vary widely with different populations and different examiners with an incidence of 1–6.6:1000 live births [5]. Identification of fetal VSD is currently considered an indication for amniocentesis, based on studies reporting that chromosomal anomalies, most commonly trisomy 21, occur in of 33%–46% [4,6,7] of fetuses with VSD. We found no studies in the literature that attempted to assess the risk of trisomy 21 when an isolated VSD, with no additional major anomalies or soft signs for Down syndrome (DS)%, was detected prenatally. This study was carried out to examine this question. 

## 2. Methods

Cases: The study group included cases in which a trained pediatric cardiologist (AN or BF) diagnosed fetal VSD between 1 January 1995 and 1 January 2007 at the fetal echocardiography clinics at Shaare Zedek and Hadassah Medical Centers. A simplified reporting system for VSD was used. The type of VSD was classified as muscular or perimembranous. This method was employed both due to occasional difficulty in making a more accurate assessment of the type of defect in the prenatal period (inlet, outlet, mal-alignment) and as the exact type of defect is more important in the pediatric patient and less so for predicting outcome in the prenatal patient. The size of the defect was assessed qualitatively. A defect was considered to be large when it was at least as large as the aortic root. It was considered small when it was slit-like or detectable only by color Doppler. Other defects were considered of medium size. This method is used, as it was felt that quantitative measurements are potentially inaccurate and add little clinically to the qualitative report. The reasons for the referral included suspected VSD on a routine anomaly scan, advanced maternal age or an otherwise high risk for aneuploidy. In all cases, anomaly scans were performed according to guidelines published by the Israeli ministry of health between 19–22 weeks and the fetal echocardiogram between 20–24 weeks of gestation. In Israel, routine anomaly scans are available, at no cost, to all patients at the mid-second trimester. 

The charts of all qualifying cases were reviewed. Exclusion criteria were maternal diabetes, teratogenic exposure, previous children with CHD, prenatal diagnosis of a major malformation, positive screen for trisomy 21 (on nuchal translucency testing (NT) with or without first trimester biochemical screening or second trimester triple testing with a calculated risk for DS greater than 1:380) or soft signs for aneuploidy and cases in which the anomaly scan report was not available for review. Nuchal fold thickness and nasal bone presence or length were not routinely measured at the time of the study. All neonates in the study group had an echocardiography by a pediatric cardiologist. Examiners performing the initial anomaly scan were all certified experts in obstetrics and gynecology with over five years of experience. 

The outcome variable was trisomy 21 in the neonate or fetus. Fetal karyotype was obtained by chart review, for patients who had amniocentesis. Microarray testing was not available during the study period, and testing for microdeletion 22q11 was not performed. When an amniocentesis was performed and the result was not noted in the chart, the patient was contacted and asked about the karyotype. To identify newborns with trisomy 21, in addition to chart review, the Israeli Ministry of Health records on neonates and abortuses with aneuploidy was crossed with the list of patients. As there is mandatory reporting of trisomy 21, this outcome would be available unless the patient delivered in another country. If not indicated in the patient’s postpartum note and the patient’s name did not appear in the Ministry of Health records, it was assumed there was no aneuploidy. 

Statistical analysis: Estimating the upper 95% CI limit was done as described by Hanley and Lippman-Hand [8]. Institutional Review Board approval was obtained for this study.

## 3. Results

We identified 126 cases with VSD and no additional cardiac anomaly. Thirty four cases were excluded: 12 had maternal diabetes, teratogenic exposure or CHD in previous children. In eight cases, anomaly scan reports were not available or the report was inadequate; two cases had additional major extra-cardiac anomalies, and 12 had “soft signs” for aneuploidy, leaving 92 cases with isolated VSD. Soft signs included pyelectasis (>4 mm), choroid plexus cyst, short femur (<5 centile) and echogenic bowel. Cases with intracardiac echogenic foci were not excluded (three cases), as we had not considered this a sign of DS*.* In 15 (16%) study group cases, chart review revealed a negative screen (NT or second trimester biochemistry). In the remaining 78, no screening test results were recorded. As seen in Table 1, the most common indication for fetal echocardiogram was suspicion of a cardiac anomaly on the routine anomaly scan. In 59 of these 63 cases, the suspicion was of VSD. In the other four, the initial suspicion was of a different cardiac anomaly. In 12 cases referred for a suspected structural problem (pericardial effusion, intra uterine growth restriction (IUGR), anomalies, soft signs), these were not confirmed on a repeat scan and, therefore, not excluded. There were four cases in the study group in which the affected fetus was one of twins.

**Table 1:** Indications for fetal echocardiogram in study group.

Indication	*N* (%)
Suspected cardiac anomaly on routine anomaly scan	63 (67)
Other suspected fetal anomalies or soft markers not confirmed on repeat scan	8 (8)
Maternal major non cardiac anomaly	6 (7)
Difficult cardiac imaging on routine scan	3 (4)
Maternal age >35	3 (4)
Abnormal triple test	3 (4)
Polyhydramnios (not confirmed on repeat scan)	2 (2)
Suspected pericardial effusion (not confirmed on echo)	1 (1)
Child with trisomy 21	1 (1)
Fetal anomaly previous pregnancy	1 (1)
IUGR% (not confirmed)	1 (1)

The mean age of cases was 30.1 (SD 5.1). Of these, 26/92 (28%) were nulliparous. 

In 74 (80.4%) cases, the VSD was described as small, in four (4.3%) cases as medium size and in one case large, and in 13 (14.1%) cases, no reference was made to VSD size. In 34 (36.9%) cases, it was muscular, in 28 (30.3%) cases membranous and in three (3.3%) cases described as “outlet”, and in 27 (29.4%) cases, no reference was made to the type of VSD. In all cases, a single defect was found. 

All patients delivered live babies, and there were no cases with trisomy 21. Trisomy 20 mosaicism was identified on amniocentesis in one case. This was assessed to be clinically insignificant; the pregnancy was continued and the child, currently eight years old, is healthy. In 60/92 (65%) cases in our study, the VSD was confirmed postpartum on echocardiography. None of the neonates had extracardiac defects on clinical examination or additional cardiac defects on echocardiography. The upper 95% CI limit for the prevalence of trisomy 21 in cases with isolated prenatal VSD is 3%; well below the reported rate for all prenatally detected VSD in the literature.

## 4. Discussion

VSD is a common anomaly with prenatal detection rates reported to be less than 40% [9,10]. Assuming a neonatal incidence of approximately 0.3%, prenatally visualized VSD would be expected to be encountered in 1:1000 pregnancies. The prognosis for VSD, both in terms of survival and quality of life, is excellent. Muscular defects frequently close spontaneously, usually in the first or second year of life. A large proportion of prenatally detected VSD’s, especially small ones, close spontaneously during intrauterine life or early neonatal life. In our study, 35% had either closed by the time of neonatal echocardiography or represented a false positive diagnosis. As this is similar to the 32%–33% reported by others [4,6], it may be suggested that our false positive rate is not excessively high. In another study [11], the closure rate was 17% (37/212) by the early neonatal period and 77% (164/212) by the age of one year. 

However, CHD in general and VSD specifically are associated with aneuploidy. Approximately one third of VSD’s in children with trisomy 21 are of the muscular type, while the majority are of the membranous and perimembranous varieties [4,12]. Up to 44% of neonates with trisomy 21 have heart defects, most commonly atrio-ventricular valve defect [5,12]. The risk for aneuploidy when CHD is diagnosed prenatally is 50%. While the association between VSD and aneuploidy was first noted in the pediatric literature, it has been observed in fetuses, as well [13,14,15]. The prevalence of trisomy in fetuses with VSD was noted to be 18.7% [16]. Attempts to extrapolate the association of VSD with aneuploidy from postnatal data to the prenatal period are all but impossible. Prenatal series are typically based on screening programs, while postnatal echocardiograms are performed for patients with features suggestive of aneuploidy or of cardiac anomalies. Technical factors render prenatal echocardiography more challenging, and detection rates might be lower and more operator dependent. Other confounding factors include the natural history of VSD, *i.e.*, the significant proportion of spontaneous closure and different proportions of fetuses with aneuploidy, depending on the type of defect. 

Previous reports of the association between VSD and various types of aneuploidy did not distinguish between isolated VSD and VSD associated with additional cardiac, as well as extra cardiac major anomalies and tended to include only small numbers of isolated VSD cases [4,6,7,10]. Specifically, no studies were designed to assess the risk for trisomy 21 when VSD is present, but no additional major anomalies or “soft” signs are seen. Given the lack of data to the contrary, many would offer karyotype examination, even for cases of isolated VSD [7,15] and possibly regardless of the result of screening results if these are available. In a recent study [11] one of 248 cases with VSD and no further anomalies had a clinically important chromosomal anomaly: A microdeletion in chromosome 22 consistent with DiGeorge syndrome. This represented 3.1% (1/32) of perimembranous defects. Despite not excluding cases with soft signs, there were no cases with trisomy in this large cohort. As microarray testing becomes commonplace, more information will be made available concerning the prevalence of microdeletions and microduplications in children and in fetuses with congenital heart disease. Sporadic cases have been reported on isolated VSD and DiGeorge syndrome [11,17]. This is not surprising given a prevalence of 14% of VSD in patients with this syndrome [18]. More research shall be required to determine a possible association between isolated prenatal VSD and abnormal chromosomal microarray results. 

This study was designed to answer a simple and common clinical question: Is the fetus with isolated VSD at risk for trisomy 21? Should fetal testing for DS be offered in these cases? This takes on special importance, as the well-described association [4,6,7,10,16] between VSD and trisomies 13 and 18 and with triploidy may not form sufficient incentive for some to offer karyotype testing, considering there are almost always associated anomalies and that these are lethal conditions. As microarray technology matures, the prevalence of microdeletions and microduplications for specific fetal anomalies, including VSD, will be the focus of future reports, which may determine the justification of genetic testing. As for all pregnant patients and until the prevalence of microarray abnormalities in patients with VSD is determined in future studies, the possibility of testing for submicroscopic chromosomal abnormalities should be discussed and offered. 

Although the proportion with a negative screen was relatively low (15/92), we could not determine with certainty the proportion of the cases with a negative screen as opposed to no DS screening at all (favoring a lower incidence of DS in our study group). We had applied exclusion criteria to avoid including cases with a clearly identifiable risk factor for VSD unrelated to DS. This might have spuriously reduced the incidence of our outcome variable. 

None of the patients in our study group had trisomy 21, and based on the study size, we conclude that the prevalence of trisomy 21 in isolated VSD cases is at most 3%. This is consistent with two previous reports which found no cases of trisomy 21 among 25 [7] and 248 [11] cases of VSD diagnosed prenatally, with no additional major anomalies. All this comes together to dispel the commonly held conviction that prenatal diagnosis of VSD is strongly associated with trisomy 21, even when the VSD is an isolated finding. We did not have data on nuchal translucency measurements (which were not routinely performed during the study period) nor on second trimester screen results (which were inconsistently performed by participants). This is because a significant proportion of our patients are orthodox Jewish or Muslim and tend to decline screening for DS. Its retrospective design and, especially, the lack of complete data on nuchal thickness or biochemical screening results for trisomy 21 are the main weaknesses of our study. An additional weakness is that although all newborns had a neonatal echocardiogram, the type of VSD was not recorded in many. Since none had trisomy 21, this does not affect our overall conclusion that a prenatally visualized VSD is not associated with a significant risk for Down syndrome.

Despite these weaknesses, we believe the study remains representative of a not uncommon clinical scenario, where an isolated VSD is diagnosed at the mid-trimester, especially when the *a priori* risk for DS according to previously performed screening procedures is small or unknown. As many, but certainly not all, patients have a first or second screening procedure, these results would apply to such a patient to which no *a priori* specific risk can be assigned or for cases in which the risk is small. The issue of genetic counseling for isolated prenatal VSD is likely to intensify in the future, due to increased machine resolution and experience of operators, as well as the wide-spread introduction of microarray testing.

We believe the major reason for the lack of association observed in this study is the inclusion of only truly isolated VSD cases, but other differences between this and previous studies may include different detection rates and the continuous improvement in the sonographic technique, leading to the detection of ever smaller and less significant VSDs. An important consideration when counseling concerns the sonographers’ ability to rule out associated anomalies and soft signs. 

## 5. Conclusions

Our results suggest that trisomy 21 is uncommon when VSD is the only sonographic abnormality. We acknowledge that an upper limit of 3% risk for DS might be too high for comfort in not suggesting amniocentesis, as is the uncertainty as to the prevalence of pathological copy number variants on chromosomal microarray. This however should be balanced against the lack of evidence in the prenatal literature between isolated VSD and DS or microarray abnormalities. Our study is underpowered to disprove an association between isolated VSD and DS, but such an association has yet to be proven in the literature. Recommendations for invasive testing in these cases should therefore be individualized, and a larger and controlled study, considering first and second trimester screening results, as well as the added risk of microarray abnormalities would be needed to determine whether the risk is low enough to preclude the need for invasive testing. 
